# Factors Affecting Health-Related Quality of Life in Patients After a Liver Transplant: A Cross-Sectional, Single-Centre, Large-Cohort Study

**DOI:** 10.3390/jcm14134507

**Published:** 2025-06-25

**Authors:** Katarzyna Kotarska, Ewa Wunsch, Jerzy Przedlacki, Maciej Wójcicki, Joanna Raszeja-Wyszomirska, Jolanta Małyszko, Piotr Milkiewicz

**Affiliations:** 1 Institute of Physical Culture Sciences, University of Szczecin, 70-453 Szczecin, Poland; katarzyna.kotarska@usz.edu.pl; 2 Department of Translational Medicine, Pomeranian Medical University in Szczecin, 71-252 Szczecin, Poland; piotr.milkiewicz@wum.edu.pl; 3 Department of Nephrology, Dialysis and Internal Medicine, Warsaw Medical University, 02-097 Warsaw, Poland; jerzy.przedlacki@wum.edu.pl (J.P.); jolanta.malyszko@wum.edu.pl (J.M.); 4 Liver and Internal Medicine Unit, Medical University of Warsaw, 02-097 Warsaw, Poland; maciej.wojcicki@wum.edu.pl (M.W.); joanna.wyszomirska@wum.edu.pl (J.R.-W.)

**Keywords:** liver transplantation, health-related quality of life, physical activity, chronic fatigue, employment

## Abstract

**Background/Objectives**: Over the past decades there has been a remarkable improvement in the clinical outcomes and prognoses after orthotopic liver transplantation (OLTx). Health-related quality of life (HRQoL) has emerged as an important concept for assessing the success of this procedure. We aimed to assess critical aspects of HRQoL in patients transplanted at our centre. **Methods**: We recruited 420 OLTx recipients and divided them into three groups based on the time since surgery. Two hundred and seventy-five controls were matched for age and gender. The Medical Outcomes Study Short Form (SF-36), International Physical Activity Questionnaire, Patient Health Questionnaire-9 (PHQ-9), and the Modified Fatigue Impact Scale (MFIS) were used to assess HRQoL, physical activity levels, depression, and chronic fatigue, respectively. **Results**: Compared to controls, OLTx patients exhibited grossly comparable HRQoL except for the physical component score (PCS) and mental domains of the SF-36. There was a significant correlation between the impairment of the PCS and depression and between the PCS and chronic fatigue. Physically active patients scored significantly lower in the MFIS, and there was a strong correlation between depression and chronic fatigue. Females exhibited more pronounced symptoms of depression and chronic fatigue than males but comparable physical activity levels and general QoL. Unemployed patients had significantly higher scores in the PHQ-9/MFIS. **Conclusions**: The general HRQoL of OLTx patients was similar to controls. However, patients were less physically active and more frequently unemployed. As depression and chronic fatigue occurred more often in females, particular attention should be paid to their psychiatric assessment.

## 1. Introduction

Orthotopic liver transplantation (OLTx) remains the only curative treatment for patients with end-stage liver disease. Currently, the five-year survival in OLTx patients exceeds 80% due to substantial improvements in managing technical issues, immunosuppression, and the management of post-transplant complications. Given these advances, issues related to patient health-related quality of life (HRQoL) and mood disturbances such as depression and chronic fatigue have emerged as pivotal for assessing the true benefit of this treatment modality [1]. Depression, in particular, may be of considerable importance, as it can not only affect patients’ compliance, but it also has a stronger negative effect on patients’ HRQoL than somatic symptoms [2]. In addition, while chronic fatigue usually decreases after the procedure, it remains in a significant proportion of patients after OLTx and, for some, may be their most distressing symptom [3,4,5].

Physical activity plays a crucial role in maintaining good health. This may be particularly important in patients who have received OLTx, as many remain sedentary with no or minimal physical activity before their operation [1]. Additionally, immunosuppressive treatments may increase various metabolic risks such as hypertension, diabetes or hyperlipidaemia. A recent study has demonstrated that a lack of physical activity leading to obesity and other metabolic problems significantly affects survival in OLTx recipients [6]. Thus, objective assessments of activity levels are essential for patient long-term survival. Returning to work should also be considered an important outcome parameter after a transplant, as it helps patients come back to everyday activities [7,8]. Unfortunately, a significant proportion of patients remain unemployed after OLTx. Currently, the literature on this subject provides conflicting data. Therefore, in this study, we aimed to assess vital factors associated with patients’ HRQoL in a large cohort of OLTx patients treated at our centre. In particular, we aimed to investigate whether the procedure has a negative impact on perceived general health by comparing scores from widely used patient-reported outcome measures (PROMs) between patients and matched controls. Additionally, we explored whether liver transplantation significantly affects levels of physical activity. Other key areas of interest included the willingness to return to work and its potential positive influence on symptoms of depression and chronic fatigue. Finally, we examined whether gender plays a role in the prevalence or severity of common symptoms impacting health-related quality of life (HRQoL).

## 2. Material and Methods

### 2.1. Patients

The study includes 420 liver transplant recipients (239 males and 181 females) under care at our unit and out-patients clinic. These patients were age- and gender-matched to 275 (138 males and 137 females) individuals with no liver disease or serious medical conditions affecting their HRQoL. Control group was recruited from patients’ families and attendees of various courses on popular science issues conducted by the authors of this manuscript. All patients and control subjects were included in the study from October 2017 until March 2020, when the study was stopped due to the COVID-19 pandemic.

All patients were enrolled in the study at least six months after undergoing transplant surgery. Study patients were clinically stable and did not suffer from severe co-morbidities that could have significantly affected their HRQoL or physical activity, such as malignant neoplasms, uncontrolled diabetes, renal failure requiring dialysis, New York Heart Association II heart failure, physical disabilities, orthopaedic or rheumatoid joint diseases, or pulmonary diseases. Patients were divided into three groups depending on the amount of time since their OLTx: 6–12 months after OLTx (76 patients [18%], group I), 13–36 months after OLTx (145 patients [35%], group II), and >37 months after OLTx (199 [47%], group III). Demographic data were collected using a self-developed questionnaire that included basic information regarding education, marital and financial status, existing addictions, and included simplified questions on general health and physical activity (Supplementary Materials S1 Questionnaire).

### 2.2. Assessment of Health-Related Quality of Life

The HRQoL was assessed using the Medical Outcomes Study Short Form (SF-36). The SF-36 is the most widely used and extensively validated generic questionnaire for assessing HRQoL in various clinical settings and populations [9]. It consists of 36 items, grouped into eight domains: physical health domains (physical functioning, role limitations due to physical problems, bodily pain, and general health) and mental health domains (vitality, social functioning, role limitations due to emotional issues, and mental health).

Each domain is scored between 0 and 100 points, with higher scores indicating better HRQoL. Two summary scores, the physical (PCS) and mental components (MCS), were obtained as a median value calculated based on the corresponding domains. This study was carried out under a licenced approval certificate (CT132326/OP012559) for the use of the SF-36 questionnaire.

### 2.3. Assessment of Physical Activity

To assess physical activity, the Polish long version of the International Physical Activity Questionnaire (IPAQ) [10] was used. IPAQ is an instrument for monitoring levels of physical activity of an adult population between 15 and 69 years of age and was developed for surveillance activities and to guide policy development related to health-enhancing physical activity across various life domains. The long version of the IPAQ comprises 27 items and investigates four physical activity domains (work, leisure, chores, and transport), as well as using time spent sitting as a proxy for sedentary behaviour [11]. Physical activity is reported as a continuous score by domain and intensity of the physical activity (moderate or vigorous) or walking. The energy cost of an activity was expressed in METs (metabolic equivalent tasks). A MET is the ratio of the energy expenditure during a given activity divided by the resting energy expenditure [10]. In this study, physical activity energy expenditure was calculated according to the following formula (in MET-min·week^−1^): number of days spent performing the activity × average duration of the activity per day × energy cost of the activity.

### 2.4. Assessment of Depressive Symptoms

The Patient Health Questionnaire-9 (PHQ-9) is a self-administered screening tool for depressive symptoms as defined in the Diagnostic and Statistical Manual IV and was initially designed as a part of the Primary Care Evaluation of Mental Disorders [12]. PHQ-9 scores range from 0 to 27 points. Scores of 5, 10, 15, and 20 points correlate with mild, moderate, moderately severe, and severe depression, respectively.

### 2.5. Assessment of Chronic Fatigue

The Modified Fatigue Impact Scale (MFIS) is a revised form of the original Fatigue Impact Scale. The Polish version was used in this study [13]. This 21-item scale provides a global score and generates three sub-scales that measure the effects of fatigue in terms of physical, cognitive and psychosocial functioning. Each item is rated by the respondent on a scale from 0 (never) to 4 (almost always). Scores are calculated for each of its three sub-scales: physical (9 items, cumulative score range 0–36), cognitive (10 items, cumulative score range 0–40), and psychosocial (2 items, cumulative score range 0–8) These scores are combined for a total MFIS score (range 0–84) [14].

### 2.6. Assessment of Bone Mineral Density

Bone mineral density (BMD) was measured in the lumbar spine (L1–L4) and total hip by dual-energy X-ray absorptiometry (GE Lunar Prodigy, Madison, WI, USA; software enCORE version 14.1) using automatic scan modes. Osteopenia and osteoporosis were defined according to World Health Organisation diagnostic criteria. BMD values were expressed in g/cm2 along with z-scores (standard deviation from the mean BMD for healthy, age-matched, gender-specific subjects) and t-scores (standard deviation from the mean BMD for young, healthy individuals).

### 2.7. Statistics

Descriptive statistical methods were used to analyse all variables. The distribution of continuous variables was visually inspected by histograms and presented as median and ranges. Categorical data are described using the number of observations and absolute frequencies. The Mann–Whitney test or the Kruskal–Wallis equality-of-populations rank test, were applied as appropriate to calculate the differences between sub-groups. Correlation analyses were performed using the Spearman rank correlation method. Prevalence comparisons between groups were performed using two-tailed Fisher’s exact test or Yates corrected Chi-squared test. Stata 18.0 (StataCorp LLC, College Station, TX, USA) was used for all calculations and graphs. A value of *p* < 0.05 was considered statistically significant.

## 3. Results

Clinical and demographic data of the patients are summarised in Table 1 and Table 2. Comparisons between the analysed patients and the control group are shown in Table 3.

All three patient groups were comparable in terms of their demographic, clinical, and health-related quality of life factors. There was no statistically significant difference between groups when the period after OLTx was analysed concerning HRQoL, physical activity, depression, or chronic fatigue (Table 1 and Table 2).

There were also no significant differences in sociodemographic variables between patients and controls except for employment, health status, and physical activity levels, as assessed using general questions from a self-prepared questionnaire (Table 3). In the SF-36, transplant patients had significantly worse HRQoL scores than controls in the domains of physical functioning, role, physical and mental health, and in the physical and mental component scores (PCS and MCS, respectively). Transplant patients were also less physically active (Table 3).

### 3.1. Correlation Between HRQoL and Physical Activity, Depression, and Chronic Fatigue

**Figure 1:**
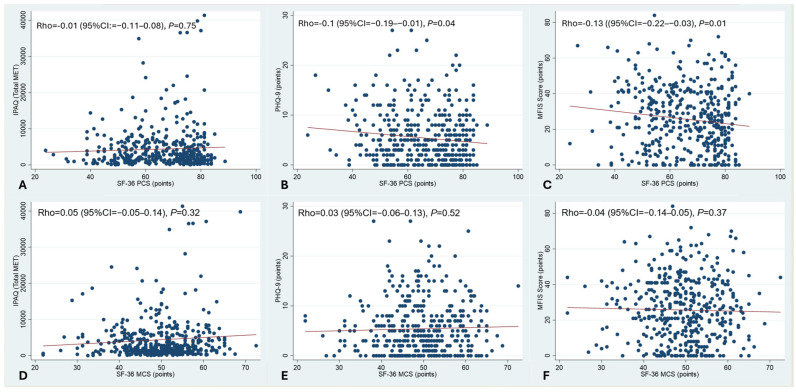
Correlation between HRQoL and physical activity, depression, and chronic fatigue. PCS and MCS were analysed. Spearman rank correlation. There was a significant correlation between the impairment of the PCS and depression, and between the PCS and chronic fatigue (**B**,**C**). No statistically significant correlation was seen between the PCS and physical activity as measured by the IPAQ (**A**). There was also no correlation between MCS and IPAQ, and depression and chronic fatigue (**D**–**F**).

For this analysis, summaries of the PCS and MCS were used. As shown in Figure 1B,C, there was a significant correlation between a pronounced impairment of the PCS and depression and between the PCS and chronic fatigue. No statistically significant correlation was seen between the PCS and physical activity as measured by the IPAQ. Additionally, there was no correlation between MCS and IPAQ, and depression and chronic fatigue. These data are summarised in Figure 1.

### 3.2. Correlation Between Physical Activity and Depression and Chronic Fatigue

**Figure 2:**
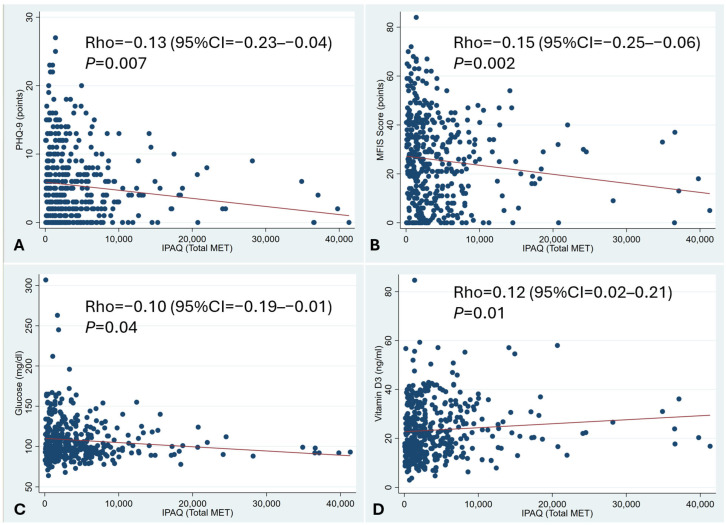
Correlation between physical activity and depression and chronic fatigue. Spearman rank correlation. A negative correlation between physical activity and depressive symptoms and between physical activity and chronic fatigue was observed (**A**,**B**). There was also a correlation between physical activity and both lower glycaemia and higher vitamin D levels (**C**,**D**).

There were negative correlations between physical activity and depressive symptoms and between physical activity and chronic fatigue (Figure 2A,B). Additionally, there was a significant correlation between physical activity and both lower glycaemia and higher vitamin D levels (Figure 2C,D).

### 3.3. Correlation Between Depression and Chronic Fatigue

**Figure 3:**
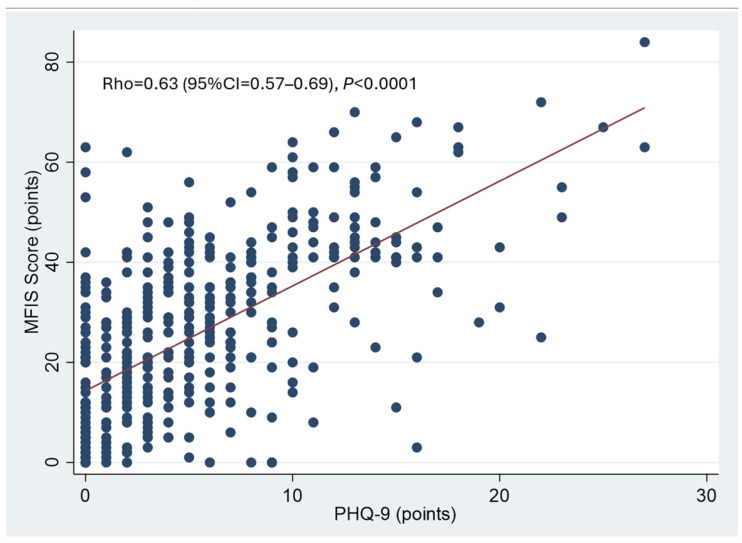
Correlation between depression and chronic fatigue. Spearman rank correlation. A significant correlation between PHQ-9 and MFIS scores was observed. Patients who were more depressed also reported more pronounced chronic fatigue.

There was a significant correlation between PHQ-9 and MFIS scores. More depressed patients complained of more pronounced chronic fatigue (Figure 3).

### 3.4. Comparison of Male and Female Patients

Female patients had significantly lower BMIs than males. They also had more pronounced symptoms of depression and chronic fatigue but comparable physical activity levels and general quality of life scores with males (Table 4).

### 3.5. Employment

Male patients were more frequently employed (65% vs. 50%, *p* = 0.004). Employed patients were also significantly younger and typically had a better PCS on the SF-36. Additionally, measures of depression and chronic fatigue were significantly better in employed than in unemployed subjects (Table 5).

### 3.6. Osteoporosis

Features of osteoporosis and osteopenia were seen in 206 (49%) patients. There was no association between bone density and post-transplant time or physical activity levels (Supplementary Table S1).

## 4. Discussion

In this study, we focused on the factors affecting the health-related quality of life of patients who underwent OLTx by analysing a large cohort of patients at our centre. To the best of our knowledge, this is the largest group of post-liver transplant patients investigated for HRQoL variables reported from a single centre. We have a long-lasting interest in various aspects of quality of life factors in patients with various liver conditions both before and after a transplant [15,16,17].

Our main findings can be summarised as follows: (i) liver transplantation does not appear to have a substantial impact on overall health-related quality of life (HRQoL); (ii) patients who have undergone liver transplantation are generally less physically active compared to carefully matched controls; (iii) transplant recipients who return to work after surgery tend to report lower levels of depression and chronic fatigue; and (iv) female patients tend to experience higher levels of depression and chronic fatigue than their male counterparts following the transplant procedure.

Indeed, when compared to age- and gender-matched controls, our patients had comparable HRQoL scores except for the PCS and MCS on the SF-36. This finding confirms the results of previous studies that have found impaired physical and mental coping strategies after surgery [18]. It has been shown that the majority of patients are more concerned about their HRQoL than longevity [18]. Hence, OLTx should aim to lead patients to a normal HRQoL, while impaired physical functioning should focus our attention on this issue and ways to improve it [19,20,21]. Of note, the patients in our study did not report more pronounced symptoms of depression or chronic fatigue compared to controls, which is in contrast to other findings [22].

It has been demonstrated that during the first six months after a liver transplant, a majority of HRQoL factors show significant improvement. Therefore, in this study, we included only patients who had undergone transplant surgery at least six months previously. We divided these patients into three groups depending on the post-surgical period, i.e., between 6 and 12 months, between 12 and 36 months, and above 37 months. These groups were comparable in terms of their clinical status and liver biochemistry. Furthermore, they did not differ regarding the analysed parameters of health-related quality of life. These results allowed us to use the entire group of transplanted patients for further analyses.

Physical activity is of particular importance in patients who undergo OLTx, as it not only improves general health but also exerts a positive effect on HRQoL [1,23,24,25]. However, an adequate methodology for the precise assessment of physical activity in transplant patients is lacking. This problem has recently been defined as a key unmet need in contemporary transplant hepatology [1]. It has been postulated that liver transplant teams should evaluate the fitness of their patients at discharge and design realistic, long-term exercise programmes aimed at achieving standards that have been established for the general population. This is of critical importance given a recent study that has highlighted the increasing proportion of physically inactive patients at risk of metabolic problems [6]. That study demonstrated that as many as 73% of transplant patients are either overweight or obese, leading to significantly increased post-transplant mortality [6]. Our study showed that higher physical activity levels correlated with lower chronic fatigue. This confirms previous findings from a study investigating a small group of patients from the Netherlands [3] and further supports the observation that physical exercise alleviates fatigue and improves QoL [5]. We also found a trend towards a correlation between physical activity and better scores in the PHQ-9, which suggests physical activity may have a protective effect against depression. This finding is in line with observations in liver transplant patients [5,22] and in non-transplant settings [26,27,28]. We also found a significant correlation between physical activity and lower glycaemia and higher vitamin D3 levels, confirming the beneficial effects of physical activity on metabolic status.

Several studies have shown that female patients with various health conditions have a worse HRQoL [29,30,31]. The reasons for this remain to be elucidated. While our study found that general HRQoL was not impaired in females after receiving a transplant, there was a striking difference regarding depression and chronic fatigue, with female patients scoring higher in both depression and chronic fatigue questionnaires than males. This finding requires further investigation, preferably with studies that involve a psychologist, to obtain further insight into this phenomenon.

The main aims of liver transplantation are to increase the length and quality of a patient’s life and enable their return to normal life, including work [8,32]. Resuming full productivity should be considered an essential outcome parameter after a solid organ transplant, as it is associated with returning to normal social life, accomplishing personal goals, and improving financial status and self-esteem [33,34]. Although the majority of liver transplants are performed on subjects of working age, a significant proportion do not return to work. Various studies have shown that between 22% and 60% of patients restart work after OLTx [35,36,37]. A large study based on United Network for Organ Sharing data, which included nearly 22,000 patients, found that only 24% were employed within 24 months after the procedure [8]. The demographic variables independently associated with employment included work activity before OLTx, age between 18 and 40 years, being male, having a college degree, and being Caucasian. Patients receiving transplants for alcoholic liver disease also had the lowest rate of employment when compared to other indications. While a detailed analysis of this issue was not the main goal of our study, we found that approximately 45% of our patients were employed. Similarly to other studies, males and younger patients were more likely to return to work. We also found that HRQoL, as measured with the SF-36, was comparable between employed and unemployed subjects. Employment also showed a highly significant correlation with lower fatigue scores and less depression in the PHQ-9. Thus, returning to work appears to have a positive effect on a patient’s mood and fatigue levels, although depression and pronounced fatigue may also preclude commencing employment.

This study has some limitations. First, this was a cross-sectional design. Second, the patients were divided into three groups based on the time after transplantation, and despite these being large cohorts, each group consisted of different patients, which may have decreased the value of our findings. Third, as the data were obtained from patient self-reporting, there may be some bias. Finally, there are no transplant-specific HRQoL questionnaires; thus, most studies, including this one, use generic forms such as the SF-36 or the PHQ-9.

In conclusion, this study showed that important markers of health-related quality of life are similar between liver transplant patients and healthy individuals, confirming that OLTx is both life-saving and offers patients an adequate HRQoL. Unfortunately, transplant patients were also found to be less physically active. Therefore, efforts should be made to encourage these patients to undertake regular exercise. Returning to work had a positive effect on patients, potentially reducing depression and chronic fatigue. As female patients were more frequently depressed and chronically fatigued, adequate counselling and, if necessary, proper treatment should be considered.

## Figures and Tables

**Table 1:** Clinical and demographic data on analysed groups of patients.

Feature	Group I (*n* = 77)	Group II (*n* = 145)	Group III (*n* = 198)	*p* Value
Age (years)	49 (21–70)	54 (21–80)	54 (21–74)	0.14
Male gender (*n*, %)	45 (58.4%)	81 (56.3%)	113 (56.8%)	0.88
BMI (kg/m^2^)	25.2 (18.2–35.5)	26.5 (17.9–41.9)	26.1 (15.6–39.0)	0.14
Aetiology (*n*, %)				0.04
Alcohol-related	18 (23.4%)	31 (21.4%)	24 (12.2%)	
Autoimmune	30 (39.0%)	41 (28.3%)	65 (33.0%)	
Viral	8 (10.3%)	39 (26.9%)	49 (24.8%)	
Other	21 (27.3%)	34 (23.4%)	59 (30.0%)	
Biochemistry				
Hb (g/dL)	13.4 (9.2–17.6)	13.9 (8.1–17.6)	14.0 (8.6–19.2)	0.03
Platelets (tys/μL)	174 (109–404)	180 (104–402)	179 (100–429)	0.93
AST (IU/L)	25 (12–319)	25 (2–505)	26 (11–555)	0.49
ALT (IU/L)	28 (10–834)	26 (10–180)	26 (5–293)	0.92
Bilirubin (mg/dL)	0.7 (0.2–5.9)	0.6 (0.2–6.0)	0.6 (0.2–9.6)	0.17
Albumin (g/dL)	4.5 (1.9–5.2)	4.6 (0.7–5.4)	4.6 (0.9–5.8)	0.04
INR	1.0 (0.3–2.4)	1.0 (0.9–2.5)	1.1 (0.7–2.9)	0.14
Glucose (mg/dL)	99 (71–245)	100 (64–212)	101 (68–307)	0.15
Vitamin D3 (ng/mL)	23.1 (7.7–84.7)	20.4 (3.0–59.3)	22.0 (3.8–58.0)	0.53
Immunosuppression TAC based (%)	71 (92%)	132 (91%)	182 (92%)	0.96
Bone density (*n*, %)				0.67
Normal	45 (58.4%)	73 (50.3%)	97 (48.7%)	
Osteopenia	28 (36.4)	64 (44.1%)	92 (46.2%)	
Osteoporosis	4 (5.2%)	8 (5.5%)	10 (5.0%)	

Group I: 6–12 months after OLTx; group II: 13–36 months after OLTx; group III: >37 months after OLTx. For continuous variables: median and range values, the Kruskal–Wallis test. For categorical data: number of observations and absolute frequencies, the Chi-square test. *Abbreviations:* ALT, alanine aminotransferase; AST, aspartate aminotransferase; BMI, body mass index; Hb, haemoglobin; INR, international normalised ratio; TAC, tacrolimus. Conversion factors to SI units are as follows: for ALT, ALP, AST, and GGT 0.0167.

**Table 2:** Comparison of patient groups in terms of analysed factors affecting health-related quality of life.

Feature	Group I (*n* = 77)	Group II (*n* = 145)	Group III (*n* = 198)	*p* Value
SF-36 PCS (points)	70 (31.25–83.75)	67.5 (23.75–88.75)	68.75 (26.5–85.0)	0.99
SF-36 MCS (points)	50 (31.25–68.75)	48.75 (26.0–63.75)	50 (21.8–72.5)	0.06
IPAQ Total MET	2316 (132–3978)	2799 (49.5–41,328)	2376 (99–37,116)	0.65
IPAQ Total sitting time (minutes/week)	2100 (21–5040)	2520 (420–5040)	2100 (14–5880)	0.81
PHQ-9 (points)	4 (0–27)	4 (0–25)	4 (0–27)	0.84
MFIS Physical (points)	12 (0–33)	12 (0–32)	11 (0–36)	0.72
MFIS Cognitive (points)	10 (0–34)	10 (0–30)	10 (0–40)	0.97
MFIS Psychosocial (points)	2 (0–8)	2 (0–8)	2 (0–8)	0.70
MFIS Total (points)	26 (0–62)	24 (0–67)	24 (0–84)	0.97

Group I: 6–12 months after OLTx; group II: 13–36 months after OLTx; group III: >37 months after OLTx. Continuous variables presented as median and range. The Kruskal–Wallis test. *Abbreviations:* IPAQ, International Physical Activity Questionnaire; MET, metabolic equivalent task; MCS, Mental Component Summary; MFIS, Modified Fatigue Impact Scale; PHQ-9, Patient Health Questionnaire-9; SF-36, Medical Outcomes Study Short Form; PCS, Physical Component Summary.

**Table 3:** Comparison of demographic and social data on analysed and control groups.

	Study Group (*n* = 420)	Control Group (*n* = 275)	*p* Value
Age (years)	53 (21–80)	52 (20–88)	0.23
Male gender (*n*, %)	239 (56.9%)	138 (50.2%)	0.10
BMI (kg/m^2^)	26.0 (15.6–41.9)	26.0 (17.2–42.9)	0.67
Employment (*n*, %)			<0.0001
No	229 (54.4%)	81 (29.5%)	
Yes	191 (45.6%)	194 (70.5%)	
Health status (*n*, %)			0.009
Poor	12 (2.8%)	8 (2.9%)	
Mild impairment	103 (24.5%)	41 (14.9%)	
Good	305 (72.7%)	226 (82.2%)	
Health-related quality of life			
SF-36 PF (points)	85.0 (0.0–100.0)	95.0 (0.0–100.0)	<0.0001
SF-36 RP (points)	75.0 (0.0–100.0)	81.5 (0.0–100.0)	0.02
SF-36 BP (points)	30.0 (0.0–90.0)	20.0 (0.0–90.0)	0.96
SF-36 GH (points)	55.0 (5.0–95.0)	55.0 (25.0–90.0)	0.89
SF-36 VT (points)	45.0 (20.0–75.0)	45.0 (20.0–80.0)	0.12
SF-36 SF (points)	50.0 (0.0–87.5)	50.0 (12.5–87.5)	0.67
SF-36 RE (points)	91.7 (0.0–100.0)	100.0 (0.0–100.0)	0.07
SF-36 MH (points)	60.0 (25.0–100.0)	65.0 (30.0–90.0)	0.007
SF-36 PCS (points)	68.7 (23.7–88.7)	72.5 (23.7–91.2)	<0.0001
SF-36 MCS (points)	50.0 (21.9–72.5)	51.2 (21.7–72.5)	0.03
PHQ-9 (points)	4 (0–27)	3 (0–27)	0.30
PHQ-9 category			0.4
Mild to moderate	222 (52.8%)	159 (57.8%)	
Moderately severe	168 (40.1%)	97 (35.3%)	
Severe	30 (7.1%)	19 (6.9%)	
MFIS Physical (points)	12 (0–36)	10 (0–36)	0.09
MFIS Cognitive (points)	10 (0–40)	10.0 (0–40)	0.86
MFIS Psychosocial (points)	2 (0–8)	2.0 (0–8)	0.63
MFIS Score (points)	25 (0–84)	23 (0–84)	0.28
Physical activity			
Physical activity			<0.0001
No	165 (39.2%)	57 (20.7%)	
Yes	160 (38.0%)	176 (64.0%)	
Time to time	96 (22.8%)	42 (15.3%)	
Total MET	2409 (49–41,328)	3012 (120–62,712)	0.02
Total sitting time (minutes/week)	2100 (14–5880)	2100 (420–5040)	0.13

For continuous variables: median and range values, the Mann–Whitney test. For categorical data: number of observations and absolute frequencies, the Chi-square test. *Abbreviations:* BP, bodily pain; GH, general health; MET, metabolic equivalent task; MCS, Mental Component Summary; MFIS, Modified Fatigue Impact Scale; MH, mental health; PHQ-9, Patient Health Questionnaire-9; SF, social functioning; SF-36, Medical Outcomes Study Short Form; PCS, Physical Component Summary; PF, physical functioning; RE, role-emotional; RP, role-physical; VT, vitality.

**Table 4:** Comparison of male and female patients with regard to analysed factors affecting health-related quality of life.

Feature	Males (*n* = 239)	Females (*n* = 181)	*p* Value
Age (years)	52.5 (21–80)	54 (21–74)	0.30
BMI (kg/m^2^)	26.7 (17.2–41.9)	25.0 (15.6–40.0)	0.004
SF-36 PCS (points)	70.0 (23.7–85.0)	67.5 (26.6–88.7)	0.72
SF-36 MCS (points)	50.0 (26.9–68.7)	50.0 (21.9–72.5)	0.79
IPAQ Total MET	2550 (66–41,328)	2376 (49–36,582)	0.45
IPAQ Total sitting time (minutes/week)	2520 (14–5580)	2100 (21–5880)	0.12
PHQ-9 (points)	3 (0–27)	5 (0–25)	<0.0001
MFIS Physical (points)	9 (0–36)	16 (0–34)	<0.0001
MFIS Cognitive (points)	7 (0–40)	13 (0–39)	<0.0001
MFIS Psychosocial (points)	2 (0–8)	3 (0–8)	<0.0001
MFIS Score (points)	20 (0–84)	32 (0–72)	<0.0001

Continuous variables presented as median and range. The Mann–Whitney test. *Abbreviations:* IPAQ, International Physical Activity Questionnaire; MET, metabolic equivalent task; MCS, Mental Component Summary; MFIS, Modified Fatigue Impact Scale; PHQ-9, Patient Health Questionnaire-9; SF-36, Medical Outcomes Study Short Form; PCS, Physical Component Summary.

**Table 5:** Comparison of employed and unemployed patients with regard to analysed factors affecting health-related quality of life.

Feature	Unemployed (*n* = 229)	Employed (*n* = 191)	*p* Value
Male gender (*n*, %)	115 (50%)	124 (65%)	0.004
Age (years)	54.3 ± 12.0	45.7 ± 11.9	<0.0001
BMI (kg/m^2^)	26.9 ± 4.7	25.8 ± 4.4	0.02
SF-36 PCS (points)	65.0 ± 13.0	67.3 ± 11.8	0.06
SF-36 MCS (points)	49.6 ± 7.6	49.4 ± 8.3	0.77
IPAQ Total MET	2140 (66–36,582)	2826 (49–41,328)	0.008
IPAQ Total sitting time (minutes/week)	2434.6 ± 1068.7	2533.0 ± 1099.3	0.36
PHQ-9 (points)	6.2 ± 5.8	4.4 ± 4.4	<0.0001
MFIS Physical (points)	13.9 ± 8.5	9.9 ± 7.5	<0.0001
MFIS Cognitive (points)	12.7 ± 8.8	8.9 ± 7.3	<0.0001
MFIS Psychosocial (points)	3.0 ± 2.2	2.1 ± 1.8	<0.0001
MFIS Score (points)	29.6 ± 18.2	20.9 ± 15.4	<0.0001

For continuous variables: median and range values, the Mann–Whitney test. For categorical data: number of observations and absolute frequencies, the Chi-square test. *Abbreviations:* IPAQ, International Physical Activity Questionnaire; MET, metabolic equivalent task; MCS, Mental Component Summary; MFIS, Modified Fatigue Impact Scale; PHQ-9, Patient Health Questionnaire-9; SF-36, Medical Outcomes Study Short Form; PCS, Physical Component Summary.

## Data Availability

Data available on request due to ethical and legal restrictions (data protection rules).

## Supplementary Materials

The following supporting information can be downloaded at https://www.mdpi.com/article/10.3390/jcm14134507/s1, Figure S1. Correlation between physical activity and depression and chronic fatigue. A negative correlation between physical activity and depressive symptoms and between physical activity and chronic fatigue was observed (1A and 1B). There was also a correlation between physical activity and both lower glycaemia and higher vitamin D levels (1C and 1D); Table S1. Comparison of patients with normal bone density, osteopenia and osteoporosis with regards to analyzed factors affecting Health Related Quality of Life Bone density; Supplementary Materials S1: Questionnaire.
