# Supplementary material for: Factors Affecting Health-Related Quality of Life in Patients After a Liver Transplant: A Cross-Sectional, Single-Centre, Large-Cohort Study
Source: J Clin Med. 2025 Jun 25;14(13):4507. doi: 10.3390/jcm14134507 (PMC12249576; doi:10.3390/jcm14134507)
Supplement: Supplementary file 1 [file jcm-14-04507-s001.zip › jcm-3649784-supplementary.pdf]

Supplementary Materials

Supplementary Materials S1. Questionnaire

I. DEMOGRAPHIC AND SOCIAL DATA

1. Gender……

2. Age………

3. Height (cm) …...............

4. Body weight (kg) ………………

5. Family situation:

□ I live alone

□ I live with my husband/wife

□ I live with my partner

□ I live with my children

□ I live with my grandchildren

□ Other, please specify: ....

6. Do you have children?

□ Yes, how many? ………………………………………………

□ No

7. Place of residence:

□ City with less than 100,000 inhabitants

□ City with more than 100,000 inhabitants

□ Village

8. Education:

□ Incomplete primary

□ Primary

□ Vocational

□ Secondary, including post-secondary

□ Higher, including Bachelor's degree

9. Are you currently employed?

□ Yes

□ No

10. If you are employed, is it:

□ Full-time

□ Part-time

□ Less than part-time

11. Type(s) of work performed:

□ Physical work

□ Mental work

□ Physical-mental work

12. Marital status:

□ Single

□ Married

□ Divorced

□ Widow/Widower

□ Civil Partnership

13. How would you describe your financial situation?

□ Luxurious (enough for everything without needing to save)

□ Very good (I live frugally, but it is enough for everything)

□ Good (I live frugally to make major purchases)

□ Adequate (I can only afford the cheapest food and clothing)

□ Rather poor (I barely make ends meet)

□ Very poor (I couldn't survive without help)

II. SELECTED HEALTH INFORMATION

14. How would you currently rate your health condition? It is:

□ Very good

□ Good

□ Fair (average)

□ Poor

□ Very poor

15. Which diseases have you been treated for, for the past 6 months or more (you may select multiple answers):

□ Hypertension

□ Diabetes

□ Ischaemic heart disease

□ Osteoporosis

□ Glaucoma

□ Hyperthyroidism

□ Depression

□ Allergic disease, specify: …………………………………………………..

□ Hemiparesis or musculoskeletal diseases

□ Alzheimer's disease

□ Other diseases, specify: ……………………………………………………

16. Do you consider yourself a sick person:

□ Yes

□ No

17. If you consider yourself a sick person, please list the chronic diseases you suffer from:

………………………………………………………………………………………

III. SELECTED LIFESTYLE INFORMATION

18. Have you ever smoked cigarettes regularly (i.e., at least one cigarette a day for a period of six months)? If yes:

□ How many years...or how many months..................................

□ No

19. I quit smoking after .... years.

20. I'm smoking now and I have been smoking for about .... years.

□ 3 - 5 times a week

□ Almost every day

21. What is the most important to you in life (underline no more than 5 answers):

□ Health

□ Family happiness

□ Motherhood/Fatherhood

□ A secure and stable job

□ A good financial situation

□ Respect from others

□ Personal independence

□ Physical fitness

□ Aesthetic appearance and clothing

□ Support from friends

□ The opportunity to explore the world

□ The opportunity for self-realisation

□ A sense of professional success

□ Other ....

22. What kind of active lifestyle do you lead?:

□ I play sports: what kind?: ………………..…. – how many times a week?: …………..

□ Walking: – one walk lasts about minutes:……….. – how many times a week?:….

□ Gardening – how many times a week?:..

□ Other activities, specify: ……………………………………………………………..

23. Do you individually engage in physical exercises (gymnastics, walking, cycling, Nordic walking, etc.)?

□ Yes

□ No

□ Sometimes

Please specify the discipline:

Supplementary Table S1. Comparison of patients with normal bone density, osteopenia and osteoporosis with regards to analyzed factors affecting Health Related Quality of Life Bone density.

Feature	Normal (N=214)	Osteopenia (N=184)	Osteoporosis (N=22)	*P* value
Age (years)	52 (21-80)	55 (21-72)	38 (21-65)	0.01
Male gender (N, %)	119 (55.4%)	109 (59.6%)	11 (50.0%)	0.56
BMI (kg/m^2)	25.2 (15.6-41.9)	27.0 (18.4-38.2)	24.2 (17.8-40.0)	0.06
Total MET	2409 (99-41328)	2346 (49-37116)	4161 (231-17220)	0.07
Total sitting time	2100 (14-5880)	2520 (420-5880)	2100 (840-4200)	0.33
SF-36 PCS (points)	70.0 (23.7-85.0)	66.8 (26.6-88.7)	67.0 (38.7-85.0)	0.56
SF-36 MCS (points)	50.0 (21.9-72.5)	49.4 (21.9-67.5)	50.4 (28.7-60.6)	0.97
PHQ-9 (points)	4 (0-27)	4 (0-23)	2 (0-25)	0.37
MFIS Physical (points)	11 (0-36)	12 (0-32)	9 (0-32)	0.75
MFIS Cognitive (points)	10 (0-40)	11 (0-34)	10.5 (0-39)	0.85
MFIS Psychosocial (points)	2 (0-8)	2 (0-8)	2 (0-6)	0.42
MFIS Score (points)	24 (0-84)	27 (0-72)	21 (0-68)	0.95

Continuous variables presented as median and range. Categorical data presented as number of observations and absolute frequencies. *Abbreviations:* IPAQ, International Physical Activity Questionnaire; MET, metabolic equivalent task; MCS, Mental Component Summary; MFIS, Modified Fatigue Impact Scale; PHQ-9, Patient Health Questionnaire-9; SF-36, Medical Outcomes Study Short Form; PCS, Physical Component Summary.

Supplementary Figure S1. Correlation between physical activity and depression and chronic fatigue. A negative correlation between physical activity and depressive symptoms and between physical activity and chronic fatigue was observed (1A and 1B). There was also a correlation between physical activity and both lower glycaemia and higher vitamin D levels (1C and 1D).

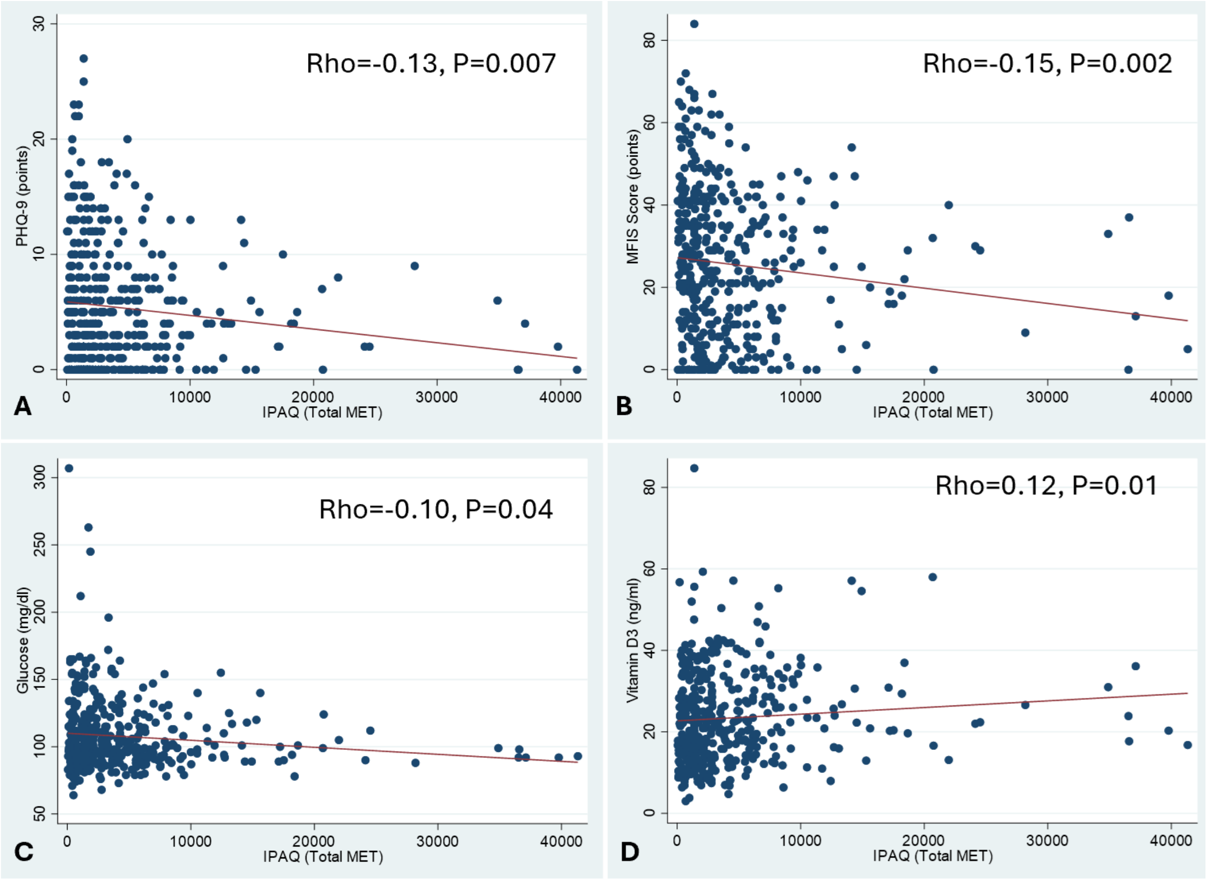
Rho=-0.13, P=0.007
PHQ-9 (points)
IPAQ (Total MET)
A
Rho=-0.15, P=0.002
MFIS Score (points)
IPAQ (Total MET)
B
Rho=-0.10, P=0.04
Glucose (mg/dl)
IPAQ (Total MET)
C
Rho=0.12, P=0.01
Vitamin D3 (ng/ml)
IPAQ (Total MET)
D
